# The impact of occasional drought periods on vegetation spread and greenhouse gas exchange in rewetted fens

**DOI:** 10.1098/rstb.2019.0685

**Published:** 2020-09-07

**Authors:** Franziska Koebsch, Pia Gottschalk, Florian Beyer, Christian Wille, Gerald Jurasinski, Torsten Sachs

**Affiliations:** 1 Landscape Ecology, University of Rostock, Rostock, Germany; 2 Geodesy and Geoinformatic, University of Rostock, Rostock, Germany; 3 GFZ German Research Centre for Geosciences, Potsdam, Germany

**Keywords:** peatland rewetting, climate extremes, founding events, gross ecosystem productivity, carry-over effects

## Abstract

Peatland rewetting aims at stopping the emissions of carbon dioxide (CO_2_) and establishing net carbon sinks. However, in times of global warming, restoration projects must increasingly deal with extreme events such as drought periods. Here, we evaluate the effect of the European summer drought 2018 on vegetation development and the exchange of methane (CH_4_) and CO_2_ in two rewetted minerotrophic fens (Hütelmoor—Hte and Zarnekow—Zrk) including potential carry-over effects in the post-drought year. Drought was a major stress factor for the established vegetation but also promoted the rapid spread of new vegetation, which will likely gain a lasting foothold in Zrk. Accordingly, drought increased not only respiratory CO_2_ losses but also photosynthetic CO_2_ uptake. Altogether, the drought reduced the net CO_2_ sink in Hte, while it stopped the persistent net CO_2_ emissions of Zrk. In addition, the drought reduced CH_4_ emissions in both fens, though this became most apparent in the post-drought year and suggests a lasting shift towards non-methanogenic organic matter decomposition. Occasional droughts can be beneficial for the restoration of the peatland carbon sink function if the newly grown vegetation increases CO_2_ sequestration in the long term. Nonetheless, care must be taken to prevent extensive peat decay.

This article is part of the theme issue ‘Impacts of the 2018 severe drought and heatwave in Europe: from site to continental scale'.

## 1. Introduction

Peatlands constitute the largest terrestrial carbon (C) stocks and exert distinct feedback mechanisms on the climate system [1,2]. The natural climate cooling effect of peatlands results from a small but persistent net sink of carbon dioxide (CO_2_) that outweighs sustained emissions of methane (CH_4_) in the long term [3]. However, intense human interference has turned global peatlands from a net sink to a source of greenhouse gases (GHG, [4]). Especially in the temperate regions of Europe, the majority of peatlands have been drained and converted to farm or forestry land. Under drainage, the peat is rapidly decomposed and substantial amounts of C are released as CO_2_. Cumulated on a global level, drained peatlands consume 10–41% of the remaining emission budget to maintain global warming below 2°C [4]. Peatland conservation and rewetting aims to reduce these emissions and is considered one of the major natural climate solutions [5,6]. In comparison to afforestation in monoculture plantations, peatland protection is expected to conserve or recreate species-rich, self-regulating ecosystems that are resilient to climate impacts [6,7].

Technically, rewetting is simply feasible by shutting down the drainage infrastructure and raising the water level. However, the preceding drainage can cause irreversible changes which constrain the ecosystem functionality of the rewetted peatland and thereby impede the achievement of the desired benefits. As peat decomposition and subsidence can lower the surface level by several decimetres [8], rewetting can create shallow lakes with water depths between 20 and 60 cm [9]. Stagnant vegetation development may retard the extensive spread of peatland species as prerequisite for CO_2_ sequestration and C accumulation [10,11]. Further, the inherent drainage-rewetting sequence may mobilize high amounts of labile C [12]. Hence, not only can rewetting create large sources of CH_4_ [13], there are also examples of rewetted peatlands with persistent high CO_2_ emissions from intense organic matter respiration [14]. Altogether, the development trajectories of rewetted peatlands and the evolving mitigation potential of rewetting measures are difficult to predict.

Under climate change, frequency and severity of hydroclimatic droughts in Central Europe are increasing [15] which poses a severe risk for peatland functioning [16] and challenges historical ecosystem conditions as targets and references of restoration measures [17]. Our knowledge on drought effects in peatlands is mostly based on research in natural boreal bogs (e.g. [18,19]) and may not necessarily apply to temperate fens. In general, the switch to aerobic conditions will likely decrease CH_4_ emissions and increase peat consumption and ecosystem respiration (Reco, [18]). Further, gross ecosystem productivity (GEP) may decrease as drought stress limits photosynthetic CO_2_ uptake [20]. Altogether, years of drought can turn peatlands from net CO_2_ sinks to sources of CO_2_ [19,20]. Although reducing CO_2_ emissions and restoring the natural C sink function are such a high priority for peatland rewetting, little is known about the consequences of hydroclimatic droughts for achieving climate mitigation goals.

Here, we aim to provide an integrated understanding of drought effects on vegetation development and the evolving GHG dynamics in rewetted minerotrophic fens and further assess the prospects of peatland rewetting as mitigation measure under climate change. To do so, we investigated the impact of the European summer drought 2018 on vegetation development, CH_4_ and net CO_2_ exchange (with its component fluxes Reco, and GEP) including possible carry-over effects in the year after the drought. The study was conducted in two degraded rewetted fens in Northeast Germany. Both sites feature comprehensive datasets on vegetation dynamics and GHG exchange dating back to 2014 so that the patterns occurring during the drought and the post-drought year can be compared against a profound reference record.

## 2. Methods

### (a) Study area

**Figure 1.**
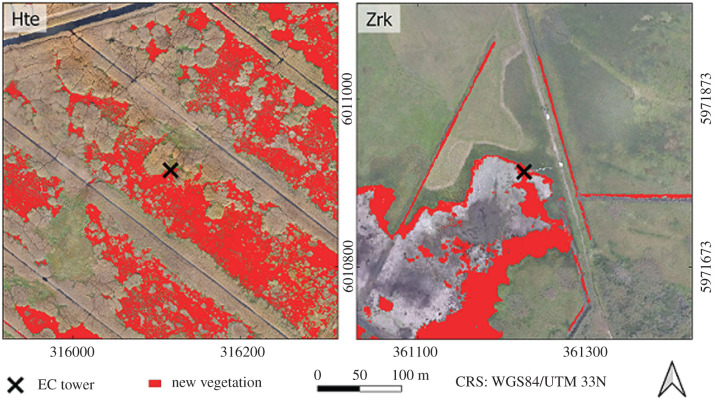
Study sites Hütelmoor (Hte) and Zarnekow (Zrk). Red colour indicates vegetation growth on former open water areas in autumn 2018. New vegetation growth was related to the last available aerial photograph before the drought, which dated back to the beginning of 2018 in Zrk and July 2015 in Hte. In the meantime, i.e. from 2015 until the start of the drought in 2018, there was no significant vegetation development in Hte.

Though the two study sites differ with respect to genesis and diagenetic processes, they share a common typology and land-use history which are typical for the fens of the northeastern German Plain. The Hütelmoor (Hte) is a coastal paludification fen located directly on the Baltic Sea (WGS84: N 54.211°, E 12.178°, figure 1). The fen features 0.2 to 2.3 m deep layers of reed-sedge peat. Hte was extensively drained for grassland use in the 1970s and gradually abandoned in the 1990s. After installation of a weir in 2010, the fen has been inundated with freshwater year-round. As a result of rewetting, the canopy turned to a small-scale mosaic of open water patches and areas vegetated with *Phragmites australis*, *Carex acutiformis*, *Bolboschoenus maritimus* and *Schoenoplectus tabernaemontani* [11]. During a storm surge in January 2019, Hte was flooded with brackish waters from the nearby Baltic Sea which complicates the interpretation of possible carry-over effects in the year after the drought.

The Polder Zarnekow (Zrk) is a fen with continuous percolating groundwater flow in the Peene River valley (53°52.50′ N, 12°53.30′ E). Peat depth partially reaches 10 m. Drainage began in the eighteenth century and was intensified between 1960 and 1990 for grassland use. Subsequent peat decomposition and surface subsidence to levels below that of the Peene River required diking and pumping, which was discontinued in the early 2000s. Starting in winter 2004/2005, the site was rewetted with water levels permanently above the ground surface. Rewetting caused an extensive dieback of the previous vegetation (mostly *Phalaris arundinacea*) and created a large shallow lake surrounded by dense dominance stands of *Typha latifolia.*

### (b) Data acquisition

CO_2_ and CH_4_ exchange rates between the atmosphere and the land surface were measured with the eddy covariance approach. This micrometeorological approach provides a continuous time series of half-hourly gas fluxes on an ecosystem scale. Though instrumentation and configuration differed slightly between both sites, each of the measurement setups is well in line with the default practice used within the eddy covariance community. Half-hourly net CO_2_ and CH_4_ fluxes were processed with the software EddyPro version 6.0.0 (LI-COR, Lincoln, NE, USA). The measured net ecosystem exchange of CO_2_ (NEE) was partitioned into the two opposed component fluxes gross ecosystem productivity (GEP, here indicated with a negative sign) and ecosystem respiration (Reco) by modelling. Reco, the CO_2_ released by autotrophic and heterotrophic respiration into the atmosphere, was obtained from night-time NEE fluxes. GEP, the photosynthetic sequestration of CO_2_ from the atmosphere into the canopy, was then calculated from the difference between NEE and Reco. The reference record to discriminate drought effects starts in 2014 when continuous measurements were available from both sites. Details of the instrumentation setup and flux processing, including gap filling and NEE partitioning, are presented in electronic supplementary material, S1. Here, we also provide further details on the acquisition of auxiliary data including (i) meteorological data, (ii) the MODIS enhanced vegetation index (EVI), which served as a proxy for plant phenology and coverage, and (iii) the classification approach to generate vegetation maps from aerial images.

## 3. Results and discussion

### (a) Meteorological conditions

Weather conditions in 2018 were characterized by a combination of persistent rain deficit and high temperatures across large parts of Europe. With only 454 mm annual precipitation at both of our study sites, 2018 was among the three driest within the last 30 years. In comparison to average years, rainfall deficits in 2018 amounted to 192 mm in Hte and to 168 mm in Zrk. Lack of rain occurred primarily from spring to summer and was most pronounced in May 2018 when precipitation hardly summed up to 5 mm (electronic supplementary material, S2). 2018 was also among the warmest two of the last 30 years with mean temperatures of 9.9°C in Hte and 9.7°C in Zrk (long-term averages: 9.3 and 9.0°C, respectively). Above-average temperatures occurred primarily during the warm season. Eventually, the combination of persistent rainfall deficit and high temperatures in 2018 culminated in a pronounced water deficit (−670 mm at Hte and −680 mm at Zrk).

### (b) Hydrological and vegetation dynamics

**Figure 2.**
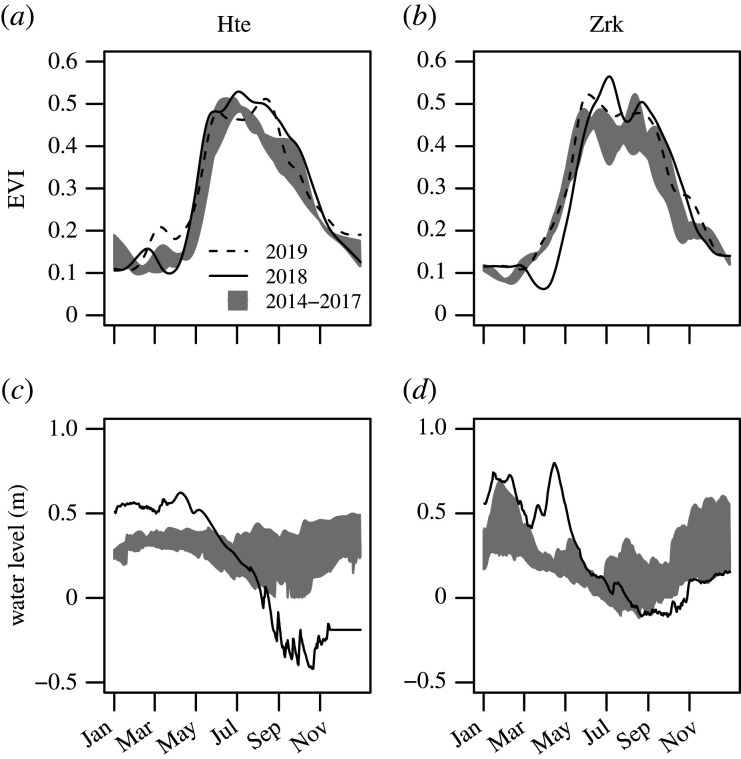
Seasonal course of the enhanced vegetation index (EVI) and water level for the year of drought 2018, the year following drought 2019 and the reference period 2014–2017. Water level time series are referenced to the average elevation height of the fens with positive values indicating water levels standing above surface. Ticks on the *x*-axis refer to the first of each month.

Both sites showed similar hydrological dynamics throughout 2018, but were to a different degree affected by drought (figure 2*a,b*). In Hte, the water level minimum was reached in September at 0.4 m below ground. At the same time, the lowering of the water table in Zrk stopped close below ground level due to water resupply from the nearby Peene river.

In both fens, the EVI peak 2018 exceeded the values of the reference records from 2014 to 2017, indicating exceptionally good growth conditions for the established vegetation in the first half of the year (figure 2*c,d*). However, as suggested by the following decline in EVI, the established vegetation began to suffer from drought stress when the spatially averaged water depth fell below 10–20 cm. The second peak in EVI occurred in late August when both sites ran completely dry, and the bare peat patches were rapidly colonized by new vegetation: in Hte, the bare flats were overgrown by *Tephroseris palustris* and *Ranunculus sceleratus*, which are pioneer plants specialized to promptly spread on nutrient-rich shores of dried-out river banks [21]. Although both species had been of negligible abundance beforehand, it took them only a few weeks to raise vegetation cover from 49 to 93%. Vegetation coverage decreased down to 60% in 2019, but was still elevated in comparison to the pre-drought condition, which was mainly due to individual occurrences of *Tephroseris palustris*. However, the interpretation of these results in Hte is complicated as possible carry-over effects of the previous drought are likely masked by the vegetation response to brackish water intrusion in January 2019. In Zrk, where the second EVI peak was more pronounced, it was *Typha latifolia*, the prevailing plant species on the spot, that transgressed previous habitat lines and thereby increased vegetation cover from 74 to 81%. Vegetation cover was still 80% in 2019, indicating that the new distribution lines of *Typha latifolia* persisted in the year after the drought.

Altogether, meteorological conditions in 2018 affected vegetation dynamics in two ways: in the first half of the year, high temperatures and radiation supply fostered exceptional growing conditions for the established vegetation until most parts of the fens fell dry and new plants, that might have been dominant before or not, took over. This pattern occurred synchronously at both study sites despite the different reference situations regarding canopy structure and species composition. Further, the massive spread of these plants is especially noteworthy as vegetation development in both sites had mostly stagnated throughout the post-rewetting period [11,14]. The observed vegetation response to drought bears substantial similarities to ecosystem dynamics of semi-permanent marsh wetlands. Here, regional climate variations induce a cyclical sequence of a shallow lake stage with little emergent vegetation and a dry stage with little standing water and dense vegetation germinating from the existing seed bank [22]. Drought periods as in summer 2018 can present a trigger event to stimulate the growth of new vegetation and close persistent gaps in the patchy canopy structure that is characteristic for many rewetted fens [10,11]. Chances are that, under otherwise constant conditions, the newly developed vegetation can be sustained beyond the actual drought period. It might, however, be advantageous if the spreading species were already predominant in the area beforehand.

### (c) Drought effects on carbon dioxide and methane exchange

**Figure 3.**
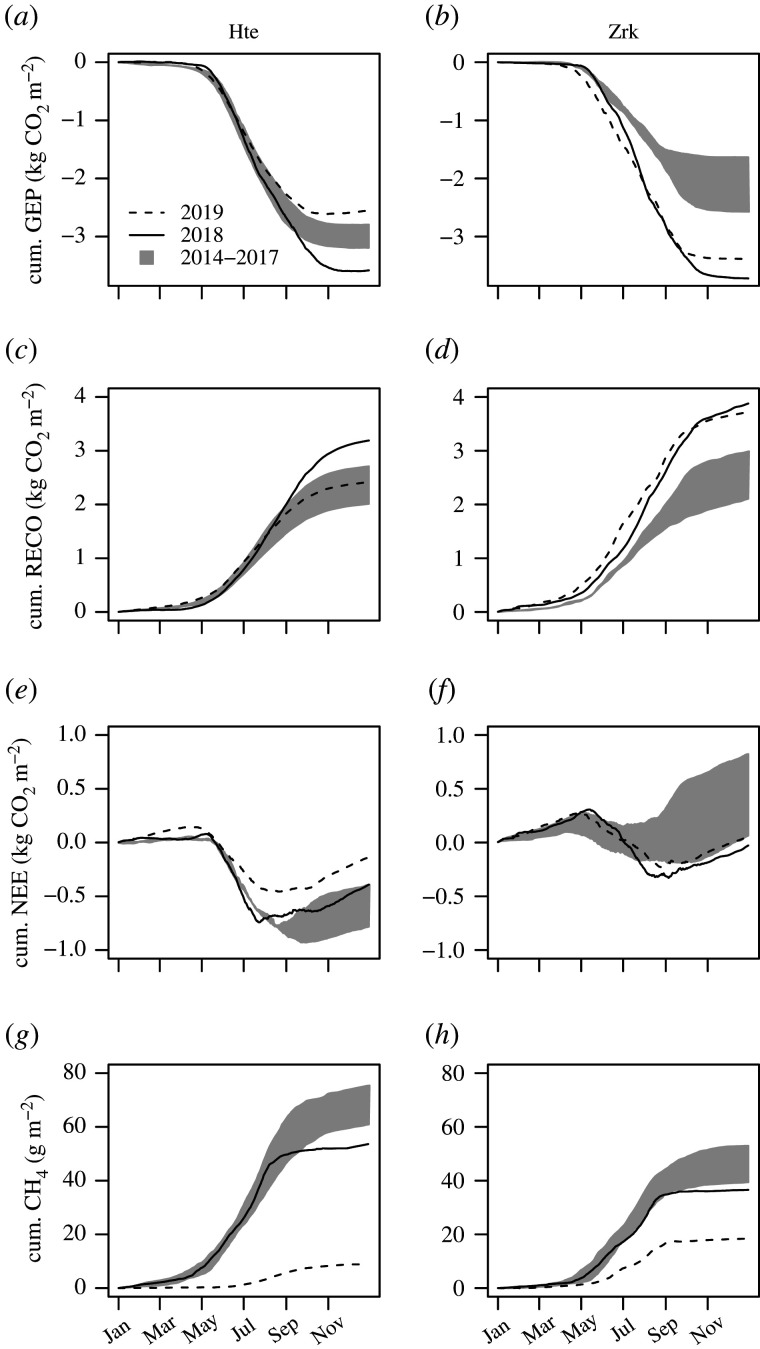
Cumulative fluxes of CO_2_ and CH_4_ for the year of drought 2018, the post-drought year 2019 and the reference period 2014–2017. Negative signs indicate CO_2_ uptake from the atmosphere into the ecosystem. Ticks on the *x*-axis refer to the first of each month.

The summer drought 2018 caused new maxima in annual GEP sums of 3.58 and 3.72 kg CO_2_ m^−2^ in Hte and Zrk (figure 3*a*,*b*). These exceeded the average annual GEP totals of the reference years 2014–2017 by 22 and 86%, respectively. Peat drought studies often report a reduction in GEP, as drought stress of plants reduces photosynthetic CO_2_ uptake [20]. Also in our study, there was a decrease in GEP in July 2018 (electronic supplementary material, S3) when the established vegetation began to suffer from drought stress. However, this decrease in GEP lasted only a few weeks at both sites. In the first half of the year, conversely, photosynthetic CO_2_ uptake was significantly higher than in reference years, as the established vegetation benefited from high radiation and high temperatures before the water finally became scarce. GEP was further boosted in the second half of the drought year, when the dried-out areas were colonized with new vegetation. In this way, very high annual GEP sums could be reached in 2018 despite drought stress of the established vegetation starting in mid growing season. In 2019, annual GEP sums in Hte decreased considerably to 2.56 kg CO_2_ m^−2^, which is probably due to the vegetation response to brackish water intrusion at the beginning of the year. Due to this event, the 2019 observations in Hte cannot be solely interpreted in relation to the previous drought period. GEP in Zrk, on the other hand, remained high in 2019, reaching annual sums of 3.39 kg m^−2^. This finding suggests that GEP remains at a high level if the new vegetation can permanently gain a foothold after drought.

The warm and dry conditions in 2018 also induced new maxima in the annual Reco sums, which amounted to 3.19 and 3.88 kg CO_2_ m^−2^ in Hte and Zrk (figure 3*c*,*d*). This corresponded to an increase of 35 and 56%, respectively, in comparison to reference years. The rise in respiratory CO_2_ release in peatlands affected by drought is generally related to peat decomposition under aerobic conditions [18]. However, given the distinct vegetation dynamics observed at our study sites, substantial parts of the high Reco sums must be attributed to increased autotrophic respiration. In Zrk, Reco remained at the same level in 2019 (3.72 kg CO_2_ m^−2^). Although high rates of autotrophic respiration can be expected again in 2019, the magnitude of the annual total suggests that heterotrophic respiration from the decomposition of organic matter will also remain high in the year after the drought.

In Hte, the resulting net CO_2_ balance amounted to −0.39 kg CO_2_ m^−2^. Hence, Hte remained a CO_2_ sink though net CO_2_ uptake was 0.18 kg m^−2^ lower than in previous years (figure 3*e*). In Zrk, photosynthetic CO_2_ sequestration almost doubled in 2018 and thereby offset respiratory CO_2_ losses by 0.03 kg CO_2_ m^−2^ (figure 3*f*). Indeed, net CO_2_ uptake was comparatively low, but the fen had previously acted as a net CO_2_ source with average NEE balances of +0.34 kg CO_2_ m^−2^ [14]. In 2019, Zrk became a net CO_2_ source of 0.06 kg m^−2^ again, though CO_2_ emissions were considerably lower than in the years before the drought.

In Hte, the annual CH_4_ sums during the drought year reached a new minimum of 53 g m^−2^ (figure 3*f*) which was 22% below the reference average. The decrease in the annual CH_4_ sums mainly concerned the period from August onwards, when fluxes were 60% lower than in the reference years (electronic supplementary material, S3). 2018 CH_4_ sums in Zrk amounted to 37 g m^−2^ which was 7 g below the average of reference years (figure 3*g*). Here, CH_4_ emissions from August onwards were 15% lower than in reference years. Although the annual CH_4_ sum was affected to varying degrees, a drought-induced reduction in CH_4_ emissions was expected since methanogenesis is bound to strictly anaerobic conditions [23].

Noteworthy, the reduction in CH_4_ emission was even stronger in the year after the drought, when annual sums decreased down to 9 and 18 g CH_4_ m^−2^ in Hte and Zrk, respectively. In Hte, we cannot discriminate the drought carry-over effect from a potential suppression of methanogenesis under high-sulfate supply [23] as it resulted from the brackish water intrusion at the beginning of 2019. The reduction of CH_4_ emissions observed in Zrk in 2019, however, can be fully attributed to the previous drought year. Incubation experiments have shown that the recovery of CH_4_ emissions following a drought can be substantially lagged due to the persistent presence of electron acceptors and/or a delayed response of the CH_4_-cycling microbial community [24]. However, studies demonstrating the full extent of drought-related after-effects on CH_4_ emissions under *in situ* conditions are rare.

In both fens, the severe drought 2018 caused common trends in CH_4_ release, GEP and Reco, whereby the effect on the net CO_2_ budget ultimately depended on the relative change in GEP compared to Reco. In addition, the year after the drought revealed marked carry-over effects in Zrk: here, the newly formed vegetation persisted under climate-normal conditions with beneficial consequences for GEP and the net CO_2_ balance. Further, drought left a lasting biogeochemical imprint that shifted organic matter decomposition towards non-methanogenic pathways but might come at the expense of increased respiratory CO_2_ release.

## 4. Conclusion

The resilience of restored peatlands to global warming is critical for future prospects of climate mitigation through peatland rewetting. Here, we report on vegetation and GHG dynamics of two rewetted temperate fens affected by the European summer drought 2018 and the post-drought year. Our study indicates common response mechanisms of rewetted fens to cope with temporal water deficit and provides wider implications for the mitigation prospects of peatland rewetting under more frequent drought occurrence.

— Practical experience has shown that the vegetation development of rewetted peatlands and the implied achievement of the mitigation goals can stagnate for years [10,11]. Drought events can overcome this stagnancy by giving impetus to the rapid spread of new vegetation. If this foundation effect comprises species that are already prevailing on the site, chances may be good that the new vegetation can also take root under climate-normal conditions. Analogies to marshes which naturally alternate between vegetated and open water stages and rely on intermittent drought to initiate vegetation development in the open water stage [22] may be helpful to better constrain climate-vegetation feedbacks in rewetted fens.
— Peatland rewetting cannot always create net CO_2_ sinks from the start [14]. The drought-induced spread in vegetation can increase photosynthetic CO_2_ sequestration and thereby move the net CO_2_ budget closer towards a sink. In addition, drought can cause a lasting biogeochemical shift towards non-methanogenic decomposition pathways, though this might come at the expense of temporary peat degradation and respiratory CO_2_ loss. Nonetheless, occasional droughts can eventually pay off if the newly established vegetation increases CO_2_ sequestration in the long term. In this regard, occasional drought events can be an integral part of fen restoration though care must be taken to prevent extensive peat decay.

## Supplementary Material

Flux processing and auxiliary data

## Data accessibility

This study is mainly based on the DE-Hte and DE-Zrk datasets available at http://www.europe-fluxdata.eu/.
