# Supplementary material for: The impact of occasional drought periods on vegetation spread and greenhouse gas exchange in rewetted fens
Source: Philos Trans R Soc Lond B Biol Sci. 2020 Sep 7;375(1810):20190685. doi: 10.1098/rstb.2019.0685 (PMC7485093; doi:10.1098/rstb.2019.0685)
Supplement: Flux processing and auxiliary data [file rstb20190685supp1.docx]

Supplementary material S1

##### CO_2_ and CH_4_ flux processing

CO_2_ and CH_4_ exchange rates between the atmosphere and the land surface were measured with the eddy covariance approach. Though instrumentation and configuration differed slightly between both sites, each of the measurement setups is well in line with the default practice used within the eddy covariance community. In Hte, the measurement setup comprised two open-path infrared gas analyzers for CO_2_ and CH_4_ molar density (LI-7500 and LI-7700, both LI-COR, Lincoln, NE, USA), and a three-dimensional sonic anemometer (CSAT3, Campbell Scientiﬁc, Logan, UT, USA) for wind velocities and sonic temperature. All signals were recorded by a CR3000 Micrologger (Campbell Scientiﬁc, Logan, Utah) with a scan rate of 10 Hz.
In Zrk, the set-up consists of an enclosed (LI-7200) and open-path (LI-7700) analyzer for CO_2_, H_2_O, and CH_4_, respectively. The sonic anemometer is a Gill HS-50 (Gill, Lymington, Hampshire, UK) and raw data were recorded with a LI-7550 digital data logger system (LI-COR Biogeosciences, Lincoln, NE, USA) at 20 Hz in half-hourly files.

Half-hourly net CO_2_ and CH_4_ ﬂuxes were processed with the software EddyPro version 6.0.0 (LI-COR, Lincoln, NE, USA) using the common corrections for open path and enclosed eddy covariance set ups. The setup and single ﬂux processing steps are described in more detail by [1, 2] for Hte and in [3] for Zrk. Data gaps in the CO_2_ ﬂux time series were ﬁlled with artiﬁcial neural networks (ANNs, [4]) based on the common back propagation algorithm incorporated in the R package neuralnet [5, 6]. Gap ﬁlling was conducted in two steps: (1) For small data gaps < 24 hours, we set up several ANNs that predicted half-hourly ﬂuxes separately for each year. (2) For larger data gaps > 24 hours, we aggregated ﬂuxes day-wise and set up a single ANN that encompassed all available measurement years 2009-2018. Input variables for all ANNs included air temperature, global radiation, and EVI, as well as fuzzy-transformed variables for time of day and season. A simple architecture comprising one hidden layer and 3-4 nodes proved applicable for all ANNs. Validation of the ANNs with an independent data subset yielded determination coeﬃcients ranging from 0.63 to 0.83 for half hourly ﬂuxes and 0.77 for daily ﬂuxes.

We partitioned NEE into its component ﬂuxes with an ANN algorithm that predicted Reco from measured night-time NEE (global radiation threshold <5 W m^-2^). Subsequently, we calculated GEP from the difference between the measured daytime NEE and modelled Reco. Input variables for the ANN included air temperature, water level, EVI, a binary rewetting indicator, as well as fuzzy-transformed variables for different seasons. The ANN was built from one hidden layer and 4 nodes. The coefficient of determination for the prediction of Reco for individual years ranged between 0.61 and 0.89 with a standard error between 0.70 and 1.32 g CO_2_ m^-2^ d^-1^ (Fig. S1-1).


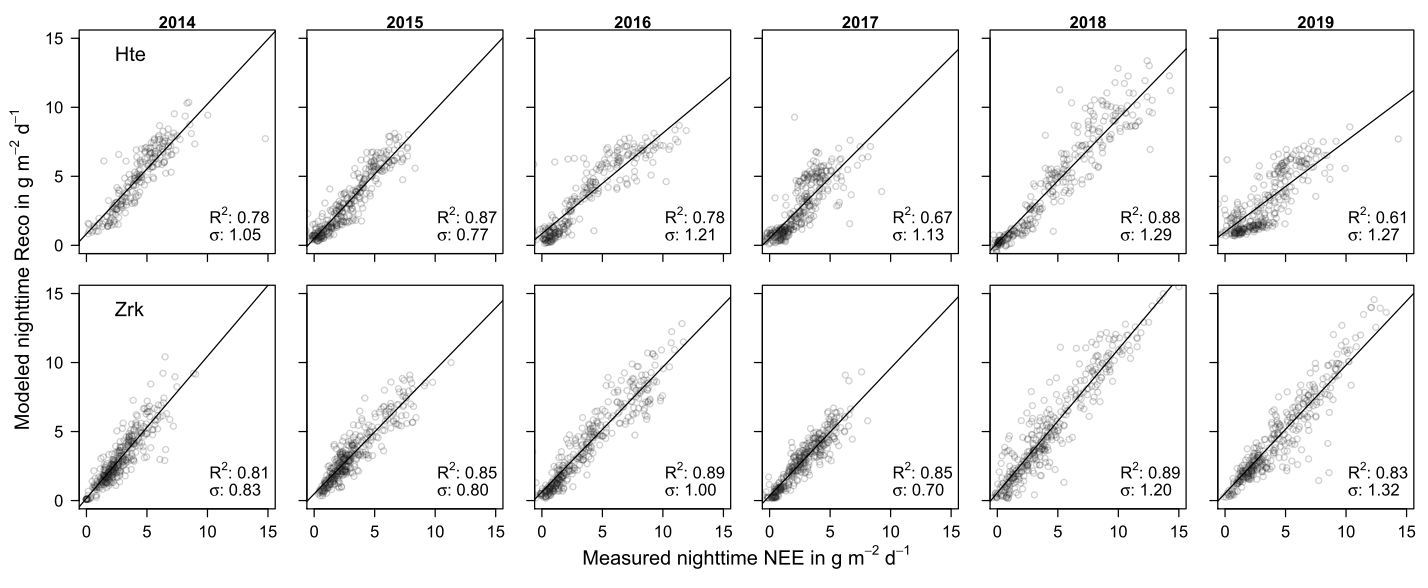


Figure S1-1 Performance of the NEE partitioning algorithm for the modelling of Reco from measured night-time NEE fluxes.

##### Auxiliary data

Meteorological data

Meteorological measurements since 2009 were conducted directly at the eddy covariance tower and logged as 30 minute averages. Measurements included (1) global radiation (Rg), measured with a pyranometer (CMP 3; Kipp & Zonen, Delft, the Netherlands), (2) air temperature (HMP45C, Vaisala, Vantaa, Finland) (3) and precipitation (52203 RM Young). Data gaps were ﬁlled with data from a nearby station of the German Weather Service (DWD,cdc.dwd.de/portal/Stations-ID: 4271). DWD weather data were also used for the meteorological long-time reference period 1999-2018.

Vegetation maps

Maps of the new vegetation that spread on former open water areas during the drought period were generated with aerial images from autumn 2018 and a reference state obtained from the last available aerial photograph before the drought. The reference image dated back to the beginning of 2018 in Zrk and to July 2015 in Hte. Our own field observations indicate that in the meantime, i.e., from 2015 until the start of the drought in 2018, there was no significant vegetation development in Hte. We used a random forest classifier with 500 trees to discriminate between vegetated and non-vegetated areas, whereby non-vegetated areas included open water and bare peat. Newly emerged vegetated areas were discerned from the intersection of the generated maps that display the reference state and the drought state, respectively.

Vegetation dynamics derived from MODIS time series

MODIS enhanced vegetation index (EVI) as proxy for plant phenology and coverage was obtained using the NASA AppEEARS tool (https://lpdaacsvc.cr.usgs.gov/appeears/). EVI values were retrieved from a 500 m x 500 m pixel (Hte) and a 250 m x 250 m pixel (Zrk) congruent with the eddy covariance ﬂux climatology, respectively. We combined data from both MODIS satellites, Aqua and Terra, and therefore obtained an EVI time series of 8 day intervals. The EVI data set was quality-ﬁltered with pixel reliability QA=0 (good data to use with confidence, Didan et al. 2015). Data gaps were subsequently ﬁlled by spline interpolation with 15 degrees of freedom.

##### References

[1] Koebsch, F., Glatzel, S., Hofmann, J., Forbrich, I. & Jurasinski, G. 2013 CO2 exchange of a temperate fen during the conversion from moderately rewetting to flooding. *Journal of Geophysical Research: Biogeosciences* **118**, 940-950.

[2] Koebsch, F., Jurasinski, G., Koch, M., Hofmann, J. & Glatzel, S. 2015 Controls for multi-scale temporal variation in ecosystem methane exchange during the growing season of a permanently inundated fen. *Agricultural and Forest Meteorology* **204**, 94-105.

[3] Franz, D., Koebsch, F., Larmanou, E., Augustin, J. & Sachs, T. 2016 High net CO2 and CH4 release at a eutrophic shallow lake on a formerly drained fen. *Biogeosciences* **13**, 3051-3070.

[4] Bishop, C. M. 1995 *Neural networks for pattern recognition*, Oxford university press.

[5] R Development Core Team. 2017 R: A Language and Environment for Statistical Computing. (Vienna, Austria, R Foundation for Statistical Computing.

[6] Fritsch, S. & Guenther, F. 2016 neuralnet: Training of Neural Networks.

Supplementary material S2


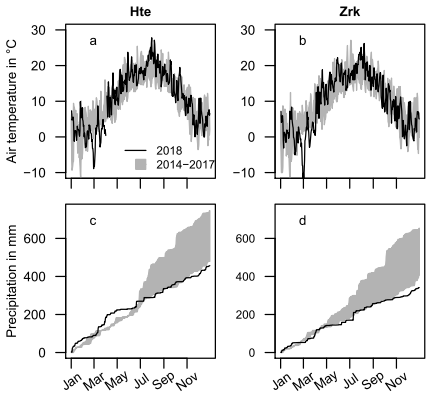


S2 Seasonal course of daily average air temperature and precipitation sums for the year of drought 2018 and the reference period 2014-2017. Ticks on the x axis refer to the first of each month.

Supplementary material S3


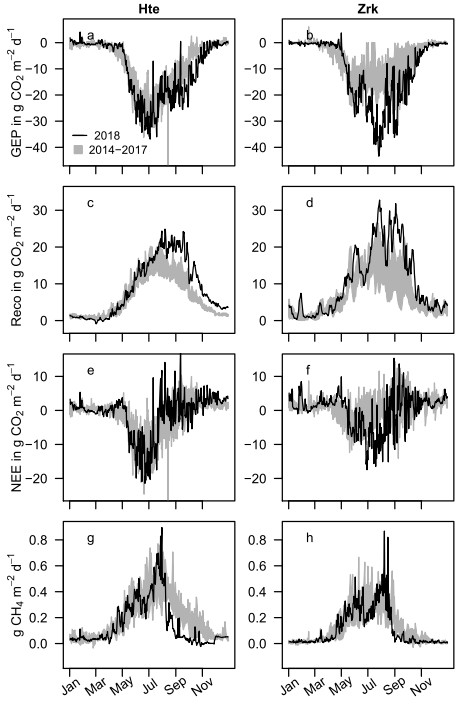


S3 Seasonal course of CO_2_ and CH_4_ fluxes for the drought year 2018 and the reference period 2014-2017. Negative signs indicate CO_2_ uptake from the atmosphere into the ecosystem. Ticks on the x axis refer to the first of each month.
